# Burden of non-communicable diseases and behavioural risk factors in Mexico: Trends and gender observational analysis

**DOI:** 10.7189/jogh.13.04054

**Published:** 2023-06-16

**Authors:** Carlos M Guerrero-López, Edson Serván-Mori, J Jaime Miranda, Stephen Jan, Emanuel Orozco-Núñez, Laura Downey, Emma Feeny, Ileana Heredia-Pi, Laura Flamand, Gustavo Nigenda, Robyn Norton

**Affiliations:** 1 Center for Health Systems Research, the National Institute of Public Health, Cuernavaca, Morelos, Mexico; 2 The George Institute for Global Health, UNSW, Sydney, Australia; 3 CRONICAS Centre of Excellence in Chronic Diseases, Universidad Peruana Cayetano Heredia, Lima, Peru; 4 The George Institute for Global Health, School of Public Health, Imperial College London, London, UK; 5 Center for International Studies, College of Mexico A.C, Mexico City, Mexico; 6 National School of Nursing and Obstetrics, the National Autonomous University of Mexico, Mexico City, Mexico

## Abstract

**Background:**

There is scarce gender-disaggregated evidence on the burden of disease (BD) worldwide and this is particularly prominent in low- and middle-income countries. The objective of this study is to compare the BD caused by non-communicable diseases (NCDs) and related risk factors by gender in Mexican adults.

**Methods:**

We retrieved disability-adjusted life years (DALYs) estimates for diabetes, cancers and neoplasms, chronic cardiovascular diseases (CVDs), chronic respiratory diseases (CRDs), and chronic kidney disease (CKD) from the Global Burden of Disease (GBD) Study from 1990-2019. Age-standardized death rates were calculated using official mortality microdata from 2000 to 2020. Then, we analysed national health surveys to depict tobacco and alcohol use and physical inactivity from 2000-2018. Women-to-men DALYs and mortality rates and prevalence ratios (WMR) were calculated as a measure of gender gap.

**Findings:**

Regarding DALYs, WMR was >1 for diabetes, cancers, and CKD in 1990, indicating a higher burden in women. WMR decreased over time in all NCDs, except for CRDs, which increased to 0.78. However, WMR was <1 for all in 2019. The mortality-WMR was >1 for diabetes and cardiovascular diseases in 2000 and <1 for the rest of the conditions. The WMR decreased in all cases, except for CRDs, which was <1 in 2020. The WMR for tobacco and alcohol use remained under 1. For physical inactivity, it was >1 and increasing.

**Conclusions:**

The gender gap has changed for selected NCDs in favour of women, except for CRDs. Women face a lower BD and are less affected by tobacco and alcohol use but face a higher risk of physical inactivity. Policymakers should consider a gendered approach for designing effective policies to reduce the burden of NCDs and health inequities.

Non-Communicable Diseases (NCDs) encompass a vast group of health conditions including cardiovascular diseases (CVDs), chronic respiratory diseases (CRDs), cancers, type 2 diabetes (T2D) and chronic kidney disease (CKD) [1]. NCDs account for over 70% of global deaths, with most of them occurring in low- and middle-income countries (LMICs) [2]. They are also responsible for 61.4% of all disability-adjusted life years (DALYs) around the world [3]. The ways people work, live and access health care system can significantly contribute to their health and protect them against death and disability. It is estimated that, in the period 2011-2030, NCDs will cost over 30 trillion US dollars (US$) worldwide, or 48% of the global gross domestic product (GDP) [2]. They impoverish households and exacerbate societal inequities. The main risk factors for developing NCDs are tobacco use, alcohol consumption, unhealthy diets, physical inactivity and air pollution [4], all modifiable through public policies [5,6].

Mexico, a LMIC of nearly 130 million inhabitants, is undergoing a demographic, epidemiological, and nutritional transition marked by growing societal inequity [7]. While the burden of disease (BD) caused by infectious diseases has decreased in some of its regions, it has remained stagnant in others. Mexico’s current epidemiological trajectory is dissonant and characterized by mounting age-specific mortality rates in adults, attributable to T2D, CKD and interpersonal violence, co-existing, however, with a relatively low burden of human immunodeficiency virus (HIV) and acquired immunodeficiency syndrome (AIDS) [7]. In 2019, NCDs amounted to 74.92% of DALYs, with an average annual change rate of 0.89% from 1990 [3].

While the importance of NCDs in Mexico has been reported [8–13], the analysis of these from a gender perspective constitutes an important gap in the literature. Biological sex imposes physiological conditions that generate heterogeneous health outcomes. In contrast, gender is a social construct entailing perceptions, norms, attitudes, and expectations regarding individuals. It is associated with occupation, education and, consequently, with income and the power relations among people. Furthermore, gender is linked to discrimination processes and determines the ways people become ill and access the health care system, particularly apparent in Mexico’s segmented health system [14]. Gender therefore acquires special relevance as a determinant of health inequity and its persistence [15].

Tackling gender disparities is a pathway to universal health coverage (UHC), one of the United Nations 2030 Sustainable Development Goals (SDGs) [16]. The Lancet has launched a Commission on Gender and Global Health [17], and the World Health Organization (WHO) has emphasized the importance of gender as a health determinant. This perspective is relevant not only to better understand the differences between men and women across various patterns of disease, but also to support the design of gender-related interventions that could effectively reduce the BD. The objective of this paper is to describe and compare the trends in the burden imposed by specific NCDs on adult women and men in Mexico as regards DALYs, mortality and behavioural risk factors. We hypothesize that the burden is differentiated by gender.

## METHODS

We conducted an observational study using several sources of information related to the burden of disease, mortality and risk factors on the population aged 20 years and older, according to the definition of adult used in the National Health and Nutrition Survey.

### Burden of disease data

We analysed data from 1990 to 2019 concerning the DALYs rates and prevalence for T2D; CKD; total cancers and neoplasms combined; and CVDs, hypertensive heart diseases, stroke and rheumatic heart diseases combined. Data were limited to the population aged 20 years and older across the 32 Mexican states, and were extracted from the GBD Study 2019 [3,18]. Detailed methods used by the GBD study are reported elsewhere [19,20]. We merged the data with the socio-demographic index (SDI) by state [21], and classified the states into SDI tertiles at 1990.

### Mortality microdata

We obtained death-register microdata for years 2000-2020 from the Mexican Statistical Office (INEGI) [22], and merged them into a single data set with more than 12.5 million death entries. We then identified those that had occurred among individuals aged ≥20 years and were attributed to the following underlying causes: T2D (ICD-10: E11-E14) [23], cancers and neoplasms (ICD-10 chapters C and D), CVDs (ICD-10: I05-I15, I60-I69), CRDs (ICD-10: J40-J44) and CKD (ICD-10: N18-N19). We calculated the age-standardized death rates for women and men aged ≥20 years, considering the age of death, year of occurrence and populations projected by the National Population Council [24], and the distribution of population according to the selected age group in 2020 as the standard population, using the direct method [25]. Finally, we identified the five most common cancers by gender. Since we used census data, no sampling assumptions were needed.

### Risk factors

Regarding risk factors, we relied on the probabilistic and population-based National Health Survey (ENSA) 2000 and the 2006, 2012, and 2018 Health and Nutrition Surveys [26]. We estimated the prevalence for tobacco use (having smoked ≥100 cigarettes lifetime and currently smoking) [27], alcohol use and daily harmful use (four or five standard drinks for women and men, respectively) [28], and physical inactivity (not having engaged in any vigorous or moderate physical activity and / or not having walked at least ten minutes continuously in the last seven days) [29]. We considered the sampling design to produce our estimates.

### Gender gap

As a measure of gender gap, we calculated the women-to-men rates ratios regarding DALYs and mortality (WMR) at *t* periods, according to the following formula:



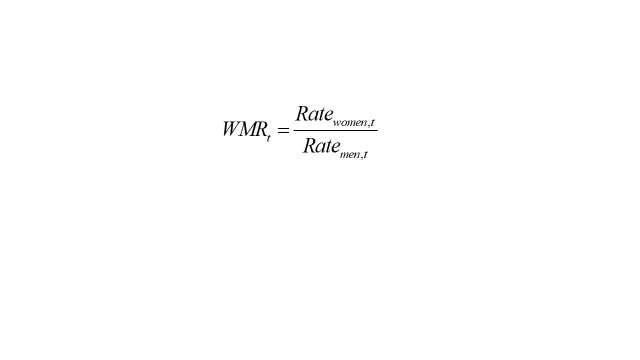



For the WMRs, the equality line is at 1, ie, the burden of outcomes is equal for women and men. When the WMR were >1, the burden was higher for women; WMR<1 implies that the burden was higher for men. We also calculated the prevalence ratios (WMR) for the risk factors.

### Analysis

To identify regional change patterns, we calculated the change rate of the DALYs and mortality the initial and final observations by state. We then described the rates for the selected diseases using multiple ordinary least squares (OLS) regressions at state level, adjusted by gender, year, interaction between gender and year, and the SDI tertile defined on the states, where first tertile corresponds to lower development and third tertile refers to more developed states according to the following:

*Y_i,t_ = β*_0_ *+ β*_1_*gender_i,t_ + β*_2_*year + β*_3_*gender_i,t_ ∙ year + β_k_SDItertile + u_i,t_*

where *i* stands for the state analysed at year *t;* gender = 1 for woman and 0 otherwise. Finally, we estimated regressions models for each SDI tertile to assess differences within the states in these groups. Statistical analyses were performed using Stata MP v17.

## RESULTS

### DALYs

**Figure 1:**
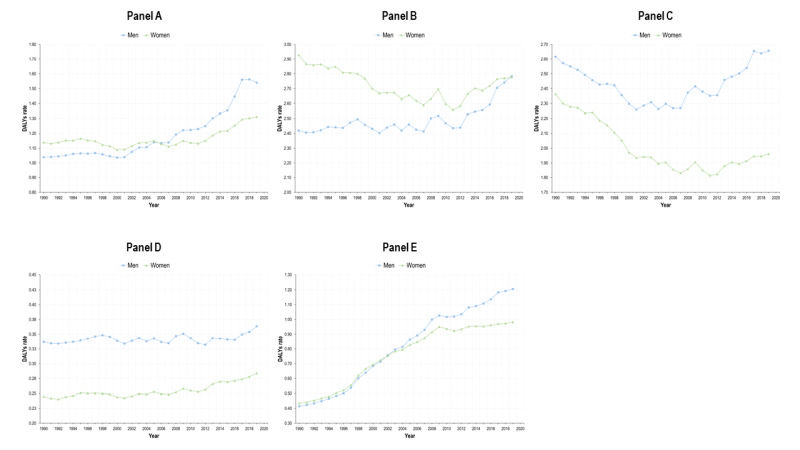
DALYs rates (per 100) for five groups of diseases, by gender, Mexico, 1990-2019. **Panel A.** Type 2 diabetes. **Panel B.** Cancers and neoplasms. **Panel C.** Chronic cardiovascular diseases. **Panel D.** Chronic respiratory diseases. **Panel E.** Chronic kidney disease.

The BD caused by NCDs showed different patterns in magnitude and trend, with heterogeneity becoming particularly prominent when gender was considered (**Figure 1**).

**Figure 2:**
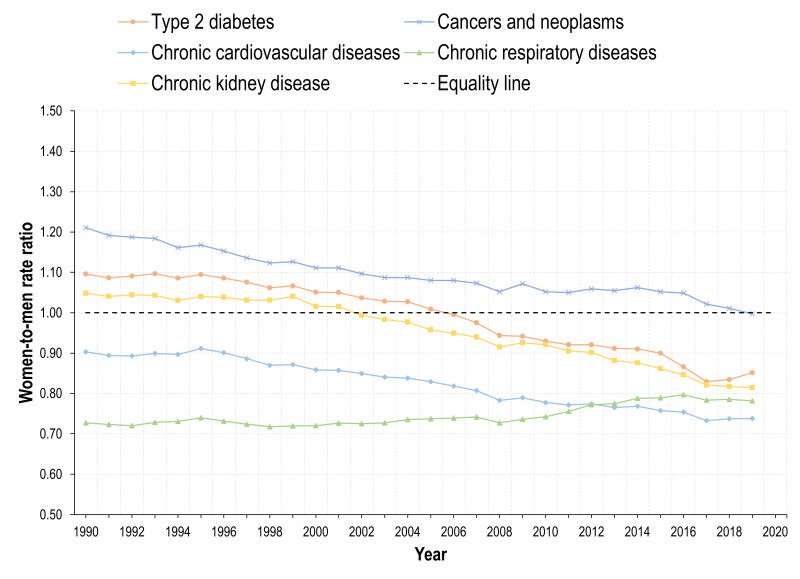
Women-to-men-rate ratios (WMRs) for DALYs caused by selected diseases. Mexico, adults 20 years+, 1990-2019.

**Figure 3:**
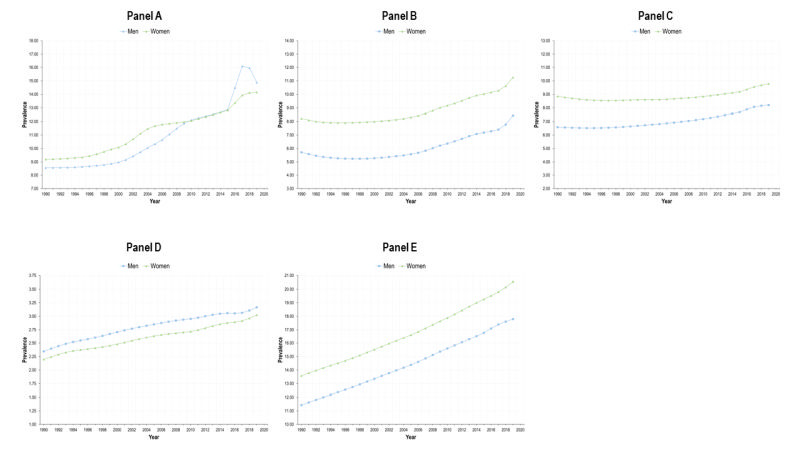
Prevalence of selected sets of diseases. Mexico, adults 20 years+, 1990-2019. **Panel A.** Type 2 diabetes. **Panel B.** Cancers and neoplasms. **Panel C.** Chronic cardiovascular diseases. **Panel D.** Chronic respiratory diseases. **Panel E.** Chronic kidney disease.

The burden caused by T2D has shown an increase by 2019, and this is more apparent for men, also with respect to trend. The trend for both genders is positive (16.3) in **Table 1** and the interaction term is negative (-12.0), which reveals that the increase is lower among women. Therefore, the DALY WMR has decreased sharply, and by 2019, is lower than 1 (**Figure 2**). The DALYs rate lines for women and men crossed around 2005. The burden caused by T2D in terms of DALYs was higher for women than for men in 1990 (an estimated difference of 170.4 DALYs / 100 000) (**Table 1**), but this burden reversed by 2019, being higher among men. States with higher SDI face a lower burden caused by diabetes (**Table 1**). The estimated national prevalence of T2D has increased steadily since 1990. In the last years, the prevalence of T2D is also higher among men (**Figure 3**).

**Table 1:** Descriptive ordinary least squares (OLS) regressions on the disability adjusted life years (DALYs) rates per 100 000 inhabitants by gender and stratified by tertile of sociodemographic index (SDI), Mexico, adults 20 y+, 1990-2019

	Outcome									
	**T2D**	***P* value**	**Cancers and neoplasms**	***P-*value**	**CVDs**	***P-*value**	**CRDs**	***P-*value**	**CKD**	***P-*value**
**Whole sample**										
Women	170.4	<0.001	388.4	<0.001	-271.8	<0.001	-111.1	<0.001	71.7	<0.001
Year	16.3	<0.001	7.3	<0.001	0.7	<0.001	0.5	0.017	28.3	<0.001
Gender x year	-12.0	<0.001	-14.3	<0.001	-14.2	<0.001	0.7	0.016	-7.6	<0.001
SDI tertile										
*1*	(ref)	(ref)	(ref)	(ref)	(ref)	(ref)	(ref)	(ref)	(ref)	(ref)
*2*	-174.1	<0.001	-10.0	0.528	-152.7	<0.001	-25.4	<0.001	-148.9	<0.001
*3*	-104.1	<0.001	273.5	<0.001	92.3	<0.001	4.5	0.205	-129.7	<0.001
Intercept	980.8	<0.001	2332.2	<0.001	2447.3	<0.001	342.9	<0.001	450.2	<0.001
**Regressions stratified by SDI tertile**										
SDI tertile 1										
*Women*	158.2	<0.001	504.6	<0.001	-156.5	<0.001	-102.6	<0.001	81.5	<0.001
*Year*	18.9	<0.001	10.1	<0.001	3.6	0.050	0.6	0.035	32.5	<0.001
*Gender x year*	-14.0	<0.001	-17.7	<0.001	-15.9	<0.001	0.8	0.042	-9.9	<0.001
*Intercept*	965.1	<0.001	2258.8	<0.001	2358.3	<0.001	336.7	<0.001	400.4	<0.001
SDI tertile 2										
*Women*	195.0	<0.001	393.1	<0.001	-261.6	<0.001	-102.3	<0.001	71.7	<0.001
*Year*	17.4	<0.001	7.9	<0.001	1.8	0.322	1.1	0.002	27.1	<0.001
*Gender x year*	-10.9	<0.001	-15.5	<0.001	-13.7	<0.001	0.4	0.383	-6.1	<0.001
*Intercept*	770.7	<0.001	2321.0	<0.001	2269.1	<0.001	306.7	<0.001	307.5	<0.001
SDI tertile 3										
*Women*	147.3	<0.001	206.7	<0.001	-461.5	<0.001	-136.9	<0.001	58.3	<0.001
*Year*	11.1	<0.001	2.4	0.273	-5.7	0.053	-0.6	0.283	24.4	<0.001
*Gender x year*	-11.0	<0.001	-7.3	0.017	-12.6	0.002	1.1	0.126	-7.1	<0.001
*Intercept*	956.8	<0.001	2717.6	<0.001	2714.6	<0.001	373.3	<0.001	379.0	<0.001

CVD – chronic cardiovascular disease, CRD – chronic respiratory disease, CKD – chronic kidney disease, SDI – sociodemographic index

There was a decrease in cancers and neoplasms among women until early 2010s, followed by a slight increase thereafter. However, a negative trend developed overall, with the coefficient for year = 7.3 and an interaction term = -14.3, resulting in an estimated trend of -7.0 among women. In contrast, the DALYs-related BD caused by cancers and neoplasms increased for men. Consequently, the WMR has also decreased from almost 1.2 to nearly 1 in 2019. In this case, the burden of disease on women was higher in 1990 (a difference of 388.4 DALYs / 100 000, constant of 2332.2 (**Table 1**) and by 2019 the WMR descended to almost the equality line. The burden of cancers and neoplasms was heterogeneous by SDI levels, with states in tertile 2 face lower burden than those in tertile 1, but states in tertile 3 had a higher burden than 1 and 2. We found a gradient showing higher DALY WMR in states with lower SDI. Prevalence of selected NCDs increased for women and men and was higher among women. However, the difference in prevalence between men and women seemed wider than differences measured in DALYs (**Figure 3**).

The DALYs burden by CVDs was consistently higher for men (WMR<1), as presented in **Figure 2****.** **Table 1** shows that the DALYs rate per 100 000 for women was 271.8 lower than men. Also, the growth was lower among women, parameter -14.2 (**Table 1**) and negative trend. Moreover, since the increase was greater for men, the WMR has been decreasing, accentuating the gender gap to the detriment of men. The difference in the trends of the burden between gender was greater to the detriment of men in lower SDI states (negative coefficient in the interaction term). The prevalence of these diseases shows the opposite pattern: women with higher prevalence than men, which contrasts with the burden of disease (**Figure 3**).

Concerning CRDs, the burden has also been greater for men between 1990 and 2019. This is reflected in the estimated parameter of -111.1 at the beginning of the period in **Table 1**. Nevertheless, the indicator shows a slight increase between 2010 and 2020, and this increase is higher among women (0.7). For that reason, the WMR has been increasing (**Figure 2**) but remains always below 1. The estimated parameters for SDI tertiles show the same patterns as in cancers and CVDs. The prevalence of CRDs is always higher for men than for women.

The burden caused by the CKD shows a consistent growth for both genders (**Table 1**). Before 2005, the WMR was around 1 in **Figure 2** but the DALYs rate for men increased more heavily than for women (**Table 1**). Therefore, by 2019 the WMR decreased sharply for CKD. As in the case of T2D, the parameters associated with SDI tertile are negative for tertiles 2 and 3, meaning that states with lower SDI faced a higher burden of disease caused by CKD compared to tertile 1. The burden is higher among men, but the prevalence is higher for women.

The trends of burden caused by all diseases included in this study varies greatly when we consider this outcome disaggregated by state, as shown in change rates in **Table 2**.

**Table 2:** Percent change in the disability adjusted life years (DALYs) rate by Mexican State, 1990-2019

State	SDI tertile	T2D		Cancers and neoplasms		CVDs		CRDs		CKD	
		**Men**	**Women**	**Men**	**Women**	**Men**	**Women**	**Men**	**Women**	**Men**	**Women**
Chiapas	1	107.0%	70.3%	15.7%	-4.9%	5.9%	-4.4%	45.8%	53.8%	206.1%	172.2%
Durango		14.0%	-0.4%	-4.2%	-9.5%	0.9%	-9.5%	-10.0%	9.0%	101.6%	82.6%
Guanajuato		47.7%	11.5%	17.9%	-6.1%	3.0%	-18.1%	6.6%	7.4%	220.1%	131.1%
Guerrero		108.3%	47.1%	42.9%	-3.6%	20.2%	-8.9%	31.7%	21.7%	199.1%	101.6%
Hidalgo		43.9%	10.7%	15.9%	-9.3%	-0.2%	-14.6%	20.2%	29.5%	190.2%	108.6%
Michoacán		65.6%	22.6%	20.0%	-13.9%	3.9%	-18.7%	14.9%	10.1%	217.3%	129.6%
Oaxaca		64.8%	31.5%	9.9%	-23.3%	-6.7%	-11.6%	23.8%	34.3%	168.1%	100.0%
Puebla		53.9%	23.3%	11.0%	-12.0%	-6.5%	-13.8%	4.0%	27.2%	201.8%	132.6%
San Luis Potosí		63.7%	19.3%	23.2%	-5.3%	2.8%	-17.0%	35.1%	34.0%	204.3%	119.6%
Tlaxcala		45.4%	7.2%	20.1%	-14.7%	-17.0%	-26.1%	4.6%	6.6%	195.5%	98.7%
Veracruz		65.2%	39.0%	26.6%	-3.0%	18.0%	-3.7%	57.1%	71.3%	250.9%	196.1%
Campeche	2	60.2%	32.5%	9.1%	-5.0%	-3.3%	-3.9%	21.9%	47.2%	187.4%	161.3%
Chihuahua		31.0%	5.5%	12.3%	1.9%	2.1%	-9.8%	7.7%	15.4%	150.1%	121.0%
Coahuila		22.1%	-7.4%	1.7%	-2.3%	3.2%	-8.4%	-22.8%	-7.6%	162.2%	144.8%
Jalisco		25.6%	-1.7%	3.5%	-16.0%	-11.4%	-33.5%	8.2%	0.4%	153.5%	76.3%
Morelos		70.6%	39.2%	19.9%	-7.0%	-2.1%	-15.6%	17.6%	37.9%	196.8%	136.4%
Nayarit		42.7%	2.7%	15.7%	-13.2%	-0.2%	-21.0%	24.9%	14.7%	188.6%	106.2%
Querétaro		33.8%	2.6%	9.4%	-2.4%	-5.5%	-19.4%	-15.3%	4.0%	168.5%	114.6%
Quintana Roo		90.9%	77.9%	19.0%	19.8%	7.8%	14.2%	34.0%	68.7%	210.3%	185.3%
Sinaloa		64.0%	15.9%	22.8%	-3.7%	24.6%	-11.1%	26.2%	20.5%	191.5%	110.7%
Tabasco		87.8%	52.5%	17.7%	-5.4%	4.0%	-12.9%	30.1%	48.5%	236.6%	194.8%
Tamaulipas		33.5%	4.9%	10.1%	-4.4%	4.0%	-14.7%	-1.6%	6.4%	195.0%	145.4%
Yucatán		46.8%	11.5%	4.6%	-16.8%	-8.0%	-16.0%	-1.4%	15.9%	144.5%	120.6%
Zacatecas		70.6%	22.1%	27.9%	-1.8%	-5.1%	-16.6%	32.1%	24.4%	202.7%	114.3%
Aguascalientes	3	20.5%	-1.2%	7.5%	-4.7%	-13.0%	-25.6%	-1.0%	9.4%	169.0%	121.4%
Baja California		17.7%	-5.8%	-3.5%	-13.3%	-16.1%	-25.8%	-4.2%	4.9%	137.7%	89.5%
Baja California Sur		50.5%	2.7%	27.2%	3.6%	21.3%	-15.5%	11.5%	9.9%	207.7%	100.1%
Colima		59.4%	5.1%	17.6%	-16.0%	-0.4%	-28.1%	14.6%	3.0%	216.7%	100.8%
México		67.1%	25.1%	29.8%	6.2%	9.0%	-17.6%	6.9%	15.6%	238.4%	143.9%
Mexico City		41.9%	8.5%	24.6%	10.5%	8.9%	-14.0%	-5.5%	10.0%	182.8%	111.0%
Nuevo León		24.8%	-5.9%	5.7%	0.0%	-6.7%	-19.8%	-19.6%	-11.3%	152.4%	107.7%
Sonora		36.5%	0.7%	0.3%	-4.2%	1.1%	-17.9%	-8.9%	16.4%	127.0%	90.3%

SDI – sociodemographic index, T2D – type 2 diabetes, CVD – chronic cardiovascular diseases, CRDs – chronic respiratory diseases, CKD – chronic kidney disease

### Mortality

**Figure 4:**
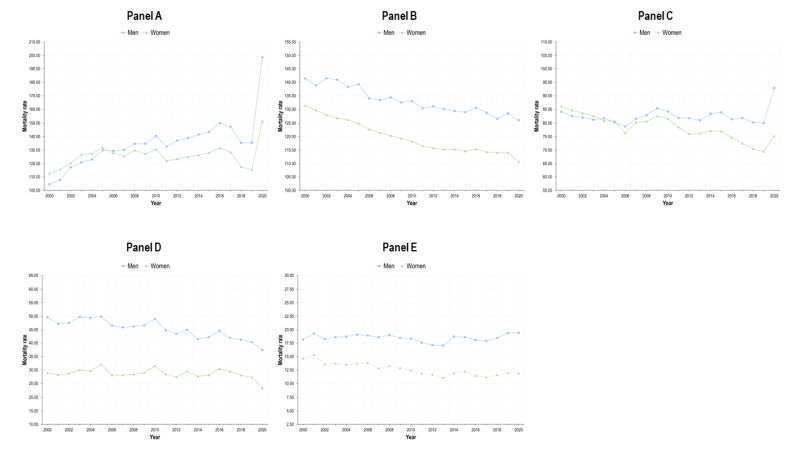
Mortality by selected non-communicable diseases (age-standardized rates per 100 000 inhabitants). Mexico, adults 20 years+, 2000-2020. **Panel A.** Type 2 diabetes. **Panel B.** Cancers and neoplasms. **Panel C.** Chronic cardiovascular diseases. **Panel D.** Chronic respiratory diseases. **Panel E.** Chronic kidney disease.

**Figure 5:**
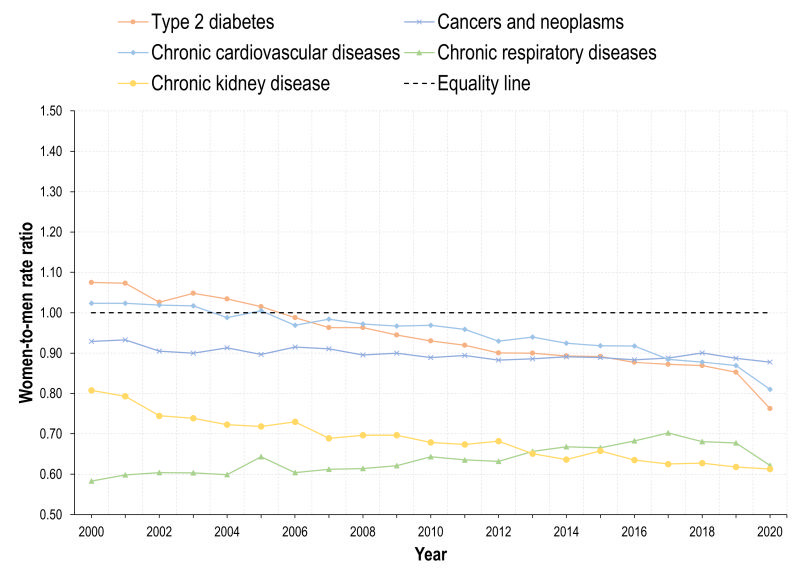
Women-to-men-rate ratios (WMRs) on Mortality, selected diseases. Mexico, adults 20 years+, 2000-2020.

Regarding T2D, we found an increase in the mortality rate both for men and women in 2020 (outbreak of COVID-19) (**Figure 4**). However, the increase was higher among men, resulting in lower mortality WMR (**Figure 5**).

**Figure 6:**
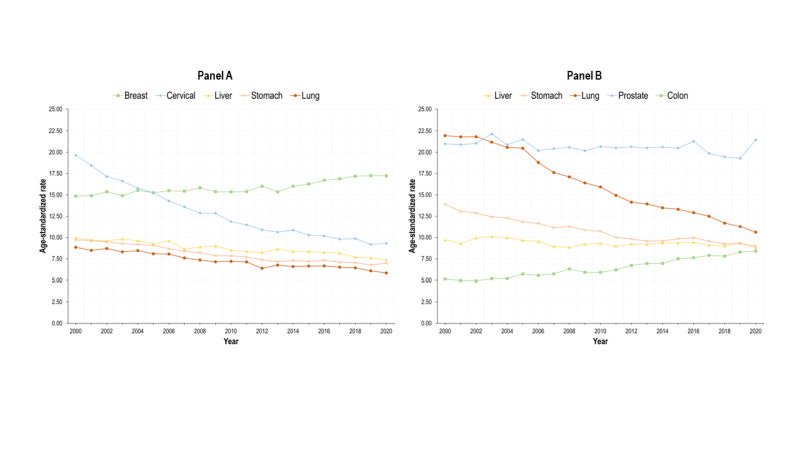
Mortality caused by main five types of cancer by gender (age-standardized rates per 100 000 inhabitants). Mexico, adults 20 years+, 2000-2020. **Panel A.** Women. **Panel B.** Men.

The deaths caused by cancers and neoplasms have increased, but the age-standardized rate has decreased nationally and for both genders. The WMR is always <1 for this set of diseases. We identified the main cancers that cause mortality disaggregated by gender (**Figure 6**). Among women, breast cancer showed a positive trend in age-standardized mortality rate, and it was the only cause that increased for them. The mortality caused by cervical cancer (the second in order of importance), liver, stomach, and lung had a negative slope during the period 2000-2020. Among men, prostate cancer was the leading cause of death among cancers and the mortality rate stagnated in the last two decades. Conversely, lung, stomach, and liver cancer showed a negative trend. In contrast, colon cancer mortality rate has been increasing steadily from 2000 to 2020 among men.

For CVDs, we found an increase both on the number of deaths and the mortality rate with an increase in 2020 for both genders. This could also be related to the COVID-19 outbreak and the reduction of health care on other diseases. However, the mortality WMR was >1 in 2000 and reverted to <1 by 2020.

The mortality caused by CRDs shows a negative trend, ie, the rate is always higher for men than for women, which implies a mortality WMR<1, but the mortality WMR has been increasing by the end of the period. The mortality rate caused by CKD shows different trends when comparing women and men. The WMR is always <1 and it is decreasing, which means that the increase is higher among men than among women.

**Table 3** shows the change rate from 2000 to 2020 by states classified according to their SDI. This reveals that the interaction between gender and place of residence yields to heterogeneous results in the mortality caused by the set of diseases considered as shown in **Table 3**.

**Table 3:** Percent change in the mortality rates by Mexican state, 2000-2020

State	SDI tertile	Diabetes		Cancers and neoplasms		CVDs		CRDs		CKD	
**Men**	**Women**	**Men**	**Women**	**Men**	**Women**	**Men**	**Women**	**Men**	**Women**
Chiapas	1	240.2%	106.8%	6.0%	-15.1%	48.7%	22.3%	30.5%	-1.4%	76.1%	22.9%
Durango		41.1%	-12.7%	-22.6%	-23.3%	-13.3%	-32.8%	-38.9%	-48.2%	-24.3%	-49.6%
Guanajuato		82.3%	28.4%	-7.4%	-10.4%	-3.4%	-22.2%	-22.2%	-19.1%	12.1%	-21.0%
Guerrero		172.0%	99.0%	-5.3%	-20.6%	36.7%	0.9%	-9.0%	-6.9%	40.5%	-3.8%
Hidalgo		102.1%	25.8%	20.1%	1.1%	1.5%	-12.7%	-11.8%	6.9%	38.4%	-27.9%
Michoacán		92.1%	26.2%	-19.1%	-18.9%	10.7%	-16.7%	-27.4%	-20.8%	12.1%	-14.2%
Oaxaca		192.3%	103.5%	6.3%	-13.1%	45.3%	7.6%	2.8%	36.1%	-0.2%	-27.5%
Puebla		136.6%	78.5%	7.0%	-7.2%	27.9%	1.2%	-0.4%	18.6%	-5.1%	-19.9%
San Luis Potosi		121.8%	52.6%	-20.6%	-12.1%	6.9%	0.9%	-0.4%	9.4%	83.3%	-16.9%
Tlaxcala		142.3%	58.3%	19.3%	14.3%	57.5%	14.4%	13.7%	27.0%	13.7%	22.7%
Veracruz		137.2%	72.5%	-9.5%	-16.5%	10.2%	-10.6%	-2.3%	-18.1%	17.2%	2.3%
Campeche	2	231.4%	101.5%	0.7%	-11.1%	47.9%	-0.2%	-1.0%	25.3%	119.4%	-31.9%
Chihuahua		65.9%	32.6%	-15.0%	-11.4%	-6.7%	-10.9%	-28.5%	-27.4%	0.3%	-6.9%
Coahuila		49.0%	5.3%	-23.8%	-21.1%	24.5%	10.1%	-48.0%	-32.2%	14.0%	-18.1%
Jalisco		30.7%	-0.2%	-8.3%	-18.5%	13.6%	-16.2%	-13.3%	-8.8%	3.2%	-15.0%
Morelos		108.4%	36.9%	5.4%	-13.8%	7.3%	-4.6%	-37.3%	-7.6%	48.5%	-12.8%
Nayarit		67.8%	9.2%	-13.1%	-19.0%	8.6%	-26.0%	-14.9%	-6.3%	-20.3%	-38.6%
Querétaro		57.0%	-8.4%	5.0%	-6.0%	-10.8%	-16.7%	-35.3%	-22.8%	52.2%	-23.6%
Quintana Roo		201.0%	124.0%	21.8%	13.6%	57.1%	11.1%	-1.0%	1.7%	65.0%	-40.0%
Sinaloa		11.0%	-9.1%	-34.1%	-26.5%	-20.3%	-39.6%	-47.2%	-44.5%	-15.4%	3.0%
Tabasco		189.5%	115.1%	-12.5%	-20.0%	24.8%	4.9%	-8.5%	-27.0%	40.8%	-15.3%
Tamaulipas		38.3%	6.2%	-29.3%	-30.3%	-6.5%	-33.1%	-37.6%	-43.2%	15.5%	-15.1%
Yucatán		104.1%	26.0%	-12.1%	-28.1%	-11.3%	-17.4%	-24.3%	17.7%	2.5%	0.0%
Zacatecas		117.1%	39.3%	-0.6%	-7.2%	-4.4%	-2.9%	-12.5%	3.3%	14.1%	14.7%
Aguascalientes	3	-14.0%	-25.8%	6.3%	-24.7%	8.1%	-24.2%	-37.5%	-43.6%	-20.9%	-49.1%
Baja California		47.9%	7.6%	-11.3%	-15.3%	5.0%	7.3%	-9.5%	-12.3%	-7.4%	-27.5%
Baja California Sur		25.5%	-23.6%	-25.1%	-7.4%	8.7%	-33.5%	-32.6%	-14.6%	-17.4%	-24.4%
Colima		73.8%	37.4%	-19.9%	2.9%	-2.8%	-32.2%	-44.4%	-49.8%	115.1%	-46.1%
Estado de México		141.0%	60.7%	-1.5%	-6.6%	28.7%	-2.0%	-30.4%	-27.9%	0.4%	-26.6%
Mexico City		47.2%	6.6%	-10.6%	-16.2%	0.7%	-27.5%	-41.2%	-30.9%	-11.0%	-31.6%
Nuevo León		43.6%	-5.9%	-21.7%	-18.6%	-6.2%	-23.7%	-50.7%	-60.0%	-38.2%	-35.7%
Sonora		23.8%	-6.2%	-22.0%	-16.5%	-4.3%	-18.7%	-34.9%	-25.5%	27.4%	-40.2%

SDI – sociodemographic index, T2D – type 2 diabetes, CVD – chronic cardiovascular disease, CRD – chronic respiratory disease, CKD – chronic kidney disease

### Risk factors

**Figure 7:**
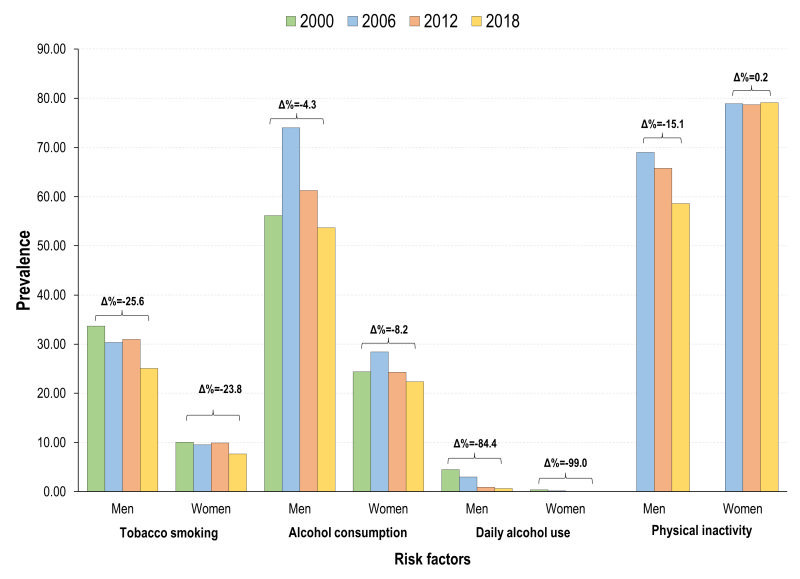
Prevalence of risk factors, by gender. Mexico, adults 20 years+, 2000-2018.

**Figure 7** shows the trends in the prevalence of risk factors for developing NCDs from 2000 to 2018. The prevalence of tobacco smoking, alcohol consumption, and daily harmful alcohol use diminished for both genders among adults and the prevalence WMR did not change significantly over the study period. However, women have a higher prevalence of physical inactivity (prevalence WMR>1) and, opposite to men, the prevalence increased over time, thus contributing to the increase in the prevalence WMR estimates.

## DISCUSSION

We show that the burden of NCDs is differentiated by gender. DALYs and mortality- caused by T2D, cancers and neoplasms, CVDs, CRDs and CKD has increased importantly in Mexico between 1990 and 2020 for men. Women face a slight reduction in cancers and neoplasms and CVDs, and increases in T2D, CKD and CRDs. In all diseases considered within these analyses, women face a lower burden of disease compared to men and are less affected by tobacco and alcohol use. However, women face an increased burden of CRDs compared to men and report a lower engagement in physical activity. The gender gap, measured by means of WMRs for DALYs and mortality rates, widened steadily for all selected NCDs in favour of women, with WMRs being <1 by the end of the study period. This held for all diseases except CRDs, for which the burden increased more for women, remaining higher, however, among men. We highlight the increased mortality by T2D and CVD in 2020, that we hypothesize was due to the COVID-19 outbreak and the associated health system crisis.

Our results are consistent with evidence reported for other regions such as Eastern Europe, where a higher BD and mortality have been registered among men than among women [30]. Our findings are also similar to the global BD by the same set of diseases according to the IHME (results not shown). When we considered socio-demographic index (SDI), we could identify areas where a greater focus on gender policy might be required. Our study substantiates the importance of monitoring subnational health trends, facilitating more efficient feedback processes for evaluating health programs and interventions. This is particularly true for states with lower SDIs, that are likely to see an increase in mortality and disability from NCDs, with catastrophic social, economic and health consequences [2].

One strength of this study resided in its use of high-quality data sources. The GBD Study provided information on a wide range of diseases and health conditions over three decades that is comparable to data from other countries in the world. The DALYs metric has the advantage of mapping premature mortality and years lived with disability into a single variable. Although the DALYs figures are estimates and there have been criticisms about its use in LMICs [31], the GBD Study is widely accepted as a powerful tool to describe the burden of disease globally. The INEGI mortality microdata also offered considerable advantages, representing the official source of information on mortality in Mexico and it has been recognized as high quality mortality data [32]. Using INEGI data afforded us to identify the specific underlying causes of death and to manage the data in a more granular way. We used nationally representative surveys to depict the trends in the prevalence of risk factors with sufficient sample sizes. Although a more recent wave of the National Health and Nutrition Survey (ENSANUT) was conducted in 2020, the sampling design, data-collection methods, and exceptional situation arising from the COVID-19 epidemic could render the latest results incomparable with those of previous rounds.

Although we did not assess formally the causal paths between variables, we believe our findings likely reflect the effects of gender norms and power imbalances prevailing in a variety of social dimensions including occupation, education, attitudes, health care seeking behaviours, risky behaviours, and political roles [33]. Concerning the Mexican health system, the current administration should accelerate the implementation of a primary-health care model, strengthen primary-care facilities with an emphasis on promotion and prevention programs by gender, expand health education for the populations at risk, and incentivize treatment adherence. The implementation of focused, effective health interventions differentiated by gender should also be encouraged, as should programs to reduce gender-based disparities and discrimination in access to health care services stemming from differentiated attitudes and limited resources. This is particularly salient in the case of CVDs and CKD, where men face a heavier burden of disease in terms of higher mortality rates, but women experience greater prevalence. These results suggest that men enjoy less access to effective health care services in Mexico than do women. Recent evidence indicates that women in Mexico are more likely than men to receive health care services when experiencing a health problem in certain contexts [34]. These disparities could constitute a major contributing factor to social inequity, yet studies on access to screening services rarely include gender-based analyses; incorporating such a perspective could help prevent the onset or worsening of NCDs [35]. Strategies to prevent and manage NCDs must address social determinants such as gender in addition to individual-level risk factors, and should be designed with the meaningful involvement of people living with NCDs.

The Mexican Health System will require additional resources that can come from strengthened fiscal policies on harmful products, such as tobacco, alcohol, sugar-sweetened-beverages and junk food, since these policies are recognized as best-buy options [36]. These policies could help to reduce the prevalence and intensity of consumption of the products that are risk factors for developing NCDs, as they can provide additional resources for health care and prevention if earmarking is strengthened. Adopting a gender transformative approach that addresses norms and power imbalances while reducing NCD risk factors and barriers to treatment would also contribute towards the United Nations Sustainable Development Goals concerning both the reduction of disability and mortality from NCDs and the achievement of gender equality [37].
